# Building metamemorial knowledge over time: insights from eye tracking about the bases of feeling-of-knowing and confidence judgments

**DOI:** 10.3389/fpsyg.2015.01206

**Published:** 2015-08-18

**Authors:** Elizabeth F. Chua, Lisa A. Solinger

**Affiliations:** ^1^Department of Psychology, Brooklyn College of the City University of New York, BrooklynNY, USA; ^2^The Graduate Center of the City University of New York, New YorkNY, USA

**Keywords:** metamemory, feeling-of-knowing, confidence, recognition, eye tracking, memory, metacognition

## Abstract

Metamemory processes depend on different factors across the learning and memory time-scale. In the laboratory, subjects are often asked to make prospective feeling-of-knowing (FOK) judgments about target retrievability, or are asked to make retrospective confidence judgments (RCJs) about the retrieved target. We examined distinct and shared contributors to metamemory judgments, and how they were built over time. Eye movements were monitored during a face-scene associative memory task. At test, participants viewed a studied scene, then rated their FOK that they would remember the associated face. This was followed by a forced choice recognition test and RCJs. FOK judgments were less accurate than RCJ judgments, showing that the addition of mnemonic experience can increase metacognitive accuracy over time. However, there was also evidence that the given FOK rating influenced RCJs. Turning to eye movements, initial analyses showed that higher cue fluency was related to both higher FOKs and higher RCJs. However, further analyses revealed that the effects of the scene cue on RCJs were mediated by FOKs. Turning to the target, increased viewing time and faster viewing of the correct associate related to higher FOKs, consistent with the idea that target accessibility is a basis of FOKs. In contrast, the amount of viewing directed to the chosen face, regardless of whether it was correct, predicted higher RCJs, suggesting that choice experience is a significant contributor RCJs. We also examined covariates of the change in RCJ rating from the FOK rating, and showed that increased and faster viewing of the chosen face predicted raising one’s confidence above one’s FOK. Taken together these results suggest that metamemory judgments should not be thought of only as distinct subjective experiences, but complex processes that interact and evolve as new psychological bases for subjective experience become available.

## Introduction

In our day-to-day functioning, we rely both on our memory, and our knowledge of our memory, referred to as our metamemory (Nelson and Narens, 1990). For example, we may encounter someone on the street, and even though we cannot immediately recall his or her name, we may feel that we know it. This feeling is the result of monitoring our memory. Because we have this feeling-of-knowing (FOK), we may continue to rack our brains for their name, showing that the knowledge of our memory influences our behavior. Once we generate a name, we then monitor and decide if we are confident enough to use that name, again showing that our knowledge of our memory influences our behavior. Memory monitoring involves evaluating the current state or ongoing progress of any aspect of memory (Nelson and Narens, 1990). In the laboratory, memory monitoring is typically measured by having individuals make subjective judgments about their memory, and can be assessed during any stage of learning and remembering. Prospective FOK judgments require participants to predict their future ability to correctly recognize some previously learned information (e.g., Shimamura and Squire, 1986; Maril et al., 2003; Perrotin et al., 2006). In contrast, *retrospective confidence judgments* (RCJs), elicited after participants have indicated their memory response, require participants to rate the likelihood that they correctly remembered the target information (e.g., Stretch and Wixted, 1998; Brewer et al., 2002; Ranganath et al., 2004; Chua et al., 2006). The typical FOK procedure uses a recall-judgment-recognition paradigm (Hart, 1965). Participants are first presented with a cue and asked to recall the corresponding target from memory. If they are unable to do so, they are asked to make a FOK judgment, followed by a recognition test. During RCJ tasks, participants are given a memory test (either recall or recognition), and subsequently asked to rate their confidence that their response is accurate.

Research has resulted in a general consensus that, when monitoring memory, individuals use an inferential process to evaluate whether a particular response will be, or has been, remembered based on the inputs that are readily available (e.g., Schwartz et al., 1997; Koriat, 2008a). However, the particular inputs that are utilized differ depending on the time at which memory is assessed (Schwartz et al., 1997). Prospective FOK judgments are thought to be based on familiarity of the cue (Metcalfe et al., 1993), accessibility of information about the target (Koriat, 1993, 1994), or a combination of the two (Koriat and Levy-Sadot, 2001). In contrast, it is widely accepted that RCJs are based on the target retrieval experience–that is, the on-line experience of remembering some previously studied item (Nelson and Narens, 1990; Chua et al., 2012; Koriat, 2012). In this study, we measured FOKs and RCJs during an associative recognition memory task, while participants had their eye movements tracked, in order to examine the sources of information that form the basis of metamemory judgments prior to, and following, recognition memory. Our goal was to examine the extent to which particular factors influence memory monitoring judgments across time.

The idea that memory monitoring is a dynamic process is central to the theoretical framework of metamemory introduced by Nelson and Narens (1990). According to the model metacognition contains two interrelated levels, an object level and a meta-level, which correspond to memory and metamemory, respectively. Information from the object level is available to the meta-level via monitoring processes, and the meta-level can modify the object level via control processes. The idea is that the meta-level contains a dynamic and imperfect model of the current state of the object level and, in the case of metamemory, monitoring is updated on-line while learning and memory processes occur. Our primary goal was to better understand this dynamic aspect of memory monitoring, and to that end, the model makes several predictions. First, we can expect that FOKs and RCJs depend on different inputs because they are made at different times over the course of retrieval. Indeed, metamemory judgments made at different points in time are often weakly correlated, implying that, at least to some extent, different information is being used as the basis of these judgments (Leonesio and Nelson, 1990; Schwartz, 1994). Secondly, although assessing memory at different times may depend on different sources of information, there may also be factors that influence the construction of the meta-model in similar ways across learning and remembering. That is, there may be sources of information that similarly influence both FOKs and RCJs. Finally, the model stipulates that monitoring informs the meta-level, which can subsequently control the object level, resulting in changes that are dynamically mapped onto the meta-level via subsequent monitoring. In other words, monitoring that takes place prior to retrieval might indirectly influence monitoring that takes place after retrieval. Therefore we can expect that FOKs may influence RCJs.

Our primary goal and novel contribution was to examine the dynamic nature of memory monitoring using eye movement indices of memory to indirectly assess the influence of available mnemonic information on FOKs and RCJs, as well as the influence of FOKs on RCJs. Although the theoretical model describes memory monitoring as a dynamic process, few studies have investigated how metamemory judgments change over time, or how multiple factors contribute to metamemory judgments. Some studies have examined how prospective judgments of learning (JOLs), another type of metamemory judgment that has participants predict their future memory performance, influenced each other in a multiple trial learning paradigm, and showed that JOLs influence each other from trial to trial (Tauber and Rhodes, 2012; Serra and Ariel, 2014), but they did not examine different types of metamemory judgments. Other studies have shown that FOKs and RCJs correlate, but did so in the service of using RCJs to index recollection or mnemonic strength to better understand FOKs (Hertzog et al., 2010, 2014), and not to understand the updating of metamemory judgments over time. We used eye movements to indirectly measure relevant factors that are known to contribute to metamemory judgments because using an indirect measure allowed us to examine several relevant factors within the same study, and on a continuous scale. This is in contrast to the majority of work investigating the basis of metamemory judgments by experimentally manipulating a single factor, and showing that the manipulation led to increases or decreases in metamemory accuracy (e.g., Begg et al., 1989; Koriat and Ma’ayan, 2005). In this study, we did not rely on a manipulation of a single factor, but instead used eye movement indices of memory to provide a sensitive measure of multiple relevant sources of information over time (Chua et al., 2012).

The first relevant factor we investigated was the cue used to elicit a target memory. Much research has focused on the influence of *cue familiarity* or *cue fluency* on FOKs (Schwartz and Metcalfe, 1992; Metcalfe et al., 1993). Increased cue familiarity has been consistently shown to lead to higher FOK ratings, even when there is no corresponding increase in memory accuracy (Reder, 1987, 1988; Schwartz and Metcalfe, 1992). This effect appears to be specific to cue familiarity because increases in target familiarity had no influence on FOKs, but did improve test performance (Schwartz and Metcalfe, 1992). Although the effects of cue fluency on FOKs have been well studied (Reder and Ritter, 1992; Schwartz and Metcalfe, 1992; Metcalfe et al., 1993; Paynter et al., 2009), less research has been devoted to considering the role of cue fluency in RCJs (but see, Diller et al., 2001). In one study, however, Chua et al. (2012) showed that increased cue familiarity led to increased RCJs. This shared basis begs the question of whether cue familiarity is an independent influence on RCJs, or whether it is mediated by FOKs. In other words, does cue familiarity influence the meta-model at the time FOK is assessed in much the same way as it does at the time RCJ is assessed, or does cue familiarity influence FOK, which then exerts a bias over the RCJ?

To measure cue familiarity, we capitalized on the ability of eye movements to indirectly measure mnemonic processing (for review, see Hannula et al., 2010). We used the *item reprocessing effect* to examine cue fluency (Althoff and Cohen, 1999; Ryan et al., 2000). Previous studies have shown that as items are processed more fluently, typically because of repeated exposure, participants make longer fixations directed to fewer regions of the picture compared to initial processing (Althoff and Cohen, 1999; Ryan et al., 2000). We have previously used eye fixations to demonstrate that cue fluency influences RCJs (Chua et al., 2012), and in this study will use it to examine both prospective FOKs and RCJs.

The next relevant factor we investigated as a basis of metamemory judgments was *partial access* to the target. FOK judgments are thought to be driven by the accessibility of information about the target (Koriat, 1993). In other words, when participants fail to recall some target information, their FOK judgments are based on the amount and fluency of partial information that is accessed while searching for the target. Indeed, partial access to various aspects of the to-be-remembered stimuli, including the emotional content (Schacter and Worling, 1985; Thomas et al., 2011), and the number of letters in a string (Koriat, 1993), led to higher FOK judgments. Further, the latency before recalling partial information has been shown to correlate with FOKs such that shorter RTs were associated with higher FOKs suggesting that the ease with which information is accessed also influences the FOK (Koriat, 1993).

To measure accessibility we relied on another eye movement based memory effect, *the rapid onset of viewing effect*. After a cue is presented, followed by a forced choice recognition task, the eyes are automatically drawn to the associated target, and this can provide an index of associative memory even independent of an explicit response (Hannula et al., 2007; Richmond and Nelson, 2009). This viewing effect has also been shown to emerge rapidly and is thought to be obligatory (Hannula et al., 2007; Ryan et al., 2007). Thus we can use rapid onset of viewing to the target as an index of memory, even if the choice differs from the target. Here we use speed of viewing directed to the target to test whether FOKs and RCJs are related to target accessibility.

The last relevant factor that we investigated as a basis of metamemory judgments is *the target recognition experience*, which has been shown to influence RCJs (Nelson and Narens, 1990; Kelley and Lindsay, 1993; Koriat and Goldsmith, 1996). Multiple components comprise the target recognition experience. For example, Kelley and Lindsay (1993) showed that confidence ratings on a general knowledge test were higher when the chosen answer had been pre-exposed, suggesting that response fluency influenced RCJs, regardless of whether it was correct or incorrect. Similarly, Chandler (1994) examined whether the amount of information accessed at retrieval influenced confidence. After studying a series of images, participants viewed a second set of images, which were similar to a subset of the studied images. During a subsequent recognition test, RCJs were higher for items from that subset even though memory performance was impaired. Finally, Busey et al. (2000) demonstrated that manipulating luminance also affects confidence, such that brighter faces (as compared to dim) lead to higher overall confidence. Taken together, these studies support the idea that the target recognition experience is often used as a basis of RCJs. Target information that is retrieved more quickly, more easily, or with more vividness is associated with higher RCJs than target information that is more effortful to retrieve (Kelley and Lindsay, 1993; Chandler, 1994; Busey et al., 2000).

The target recognition experience can also be studied using eye movements in forced choice paradigms (Hannula and Ranganath, 2009; Chua et al., 2012). Previous research has shown a *disproportionate viewing effect* in that participants look longer at the studied stimulus compared to the non-studied distractors (Hannula et al., 2007; Ryan et al., 2007). Proportion of viewing among the different choices can be examined in relation to the correct face (which is chosen for hits and not misses), and also to the chosen face (which is correct for hits and not misses). Disproportionate viewing of the correctly chosen target is a memory effect that occurs above and beyond viewing related to choice (Hannula et al., 2007), and thus can be used to index retrieval. Additionally, increased viewing of the chosen stimulus has been related to choice certainty (Chua et al., 2012). Thus, we use disproportionate viewing to examine how the recognition experience relates to metamemory judgments.

In our study, to examine the basis of memory monitoring at differing points across the learning and memory time scale, subjects completed a face-scene associative memory task while having their eye movements monitored (Hannula et al., 2007; Hannula and Ranganath, 2009; Chua et al., 2012). After studying face-scene pairs, participants were presented with a scene cue, and then rated their FOK that they would remember the associated face. This was followed by a forced choice recognition test for the face that had been studied with the scene and retrospective confidence ratings. Using this paradigm we tested whether eye movement measures of cue fluency, accessibility, and the recognition experience contributed to FOKs and RCJs. We further tested whether relevant factors, namely cue fluency, had a direct influence on RCJs or were mediated by FOKs.

## Materials and Methods

### Participants

Seventy English speaking Brooklyn College students participated in this research in exchange for course credit (1credit/h) or for pay ($10/h). All participants reported normal or corrected to normal vision. Only data from 66 participants (47 females/19 males; mean age = 21, range 18–34 years) were analyzed; data from other participants were excluded because eye position could not be reliably calibrated, or time constraints and technical difficulties did not allow for sufficient data to be collected. Each participant provided written informed consent in a manner approved by the Human Research Protection Program at Brooklyn College.

### Behavioral Paradigm

PsychoPy software was used to present stimuli and record responses (version 1.81; http://www.psychopy.org; Peirce, 2007). Participants viewed stimuli on a secondary 22″ monitor with an integrated eye tracking camera unit controlled by a Windows PC.

Stimuli consisted of 180 full-color face images (90 females/90 males) selected from a previously normed faces database (Althoff and Cohen, 1999) and 180 full-color scenes from Brand X© photography. Each face was sized to 256 × 256 pixels and placed upon a 270 × 270 pixels uniform black background; scenes were sized to 800 × 600 pixels. The size of the face displayed on the monitor was 9.7 cm × 9.7 cm, and the size of the scenes on the display was 29.6 cm × 22.2 cm. Nine additional face images and three additional scene images were used for instruction and practice of the behavioral paradigm.

**FIGURE 1:**
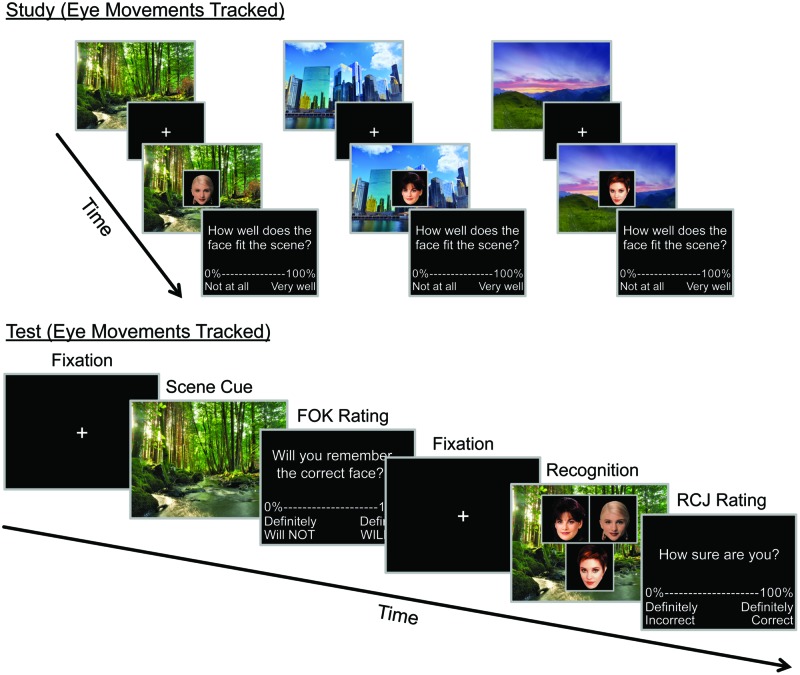
**Behavioral Paradigm.** Example of three study trials and one cued recognition test trial. During the three-face test display participants made a three-alternative forced choice recognition decision to indicate which face had been previously paired with the scene. The duration of each trial component at test is displayed below the corresponding image.

Data were obtained in five blocks, each of which comprised of an encoding phase and a self-paced three alternative forced choice recognition (3AFC) test phase that also included assessments of FOK and confidence (**Figure 1**). Of the 66 participants included in the analyses, 39 completed all five blocks while 19 completed four blocks due to time constraints. Each study phase consisted of encoding 36 face-scene pairs (18 female/18 male). The face-scene combinations were chosen randomly and randomly assigned to a specific block. The presentation order of the blocks, as well as the trials within each block, was independently randomized. Each study trial began by presenting a scene on the screen for 3000 ms, followed by a gaze-contingent fixation cross for 500 ms, and then a face appeared that was superimposed on the scene for 4000 ms. To ensure that participants attended to each face-scene pair, participants were instructed to rate how well they thought the face “fit” the scene, using the 11 keys across the top of the keyboard (from the symbol ∼ to the number 0) to indicate responses from 0 to 100 in intervals of 10. Participants were also instructed to try to remember each face-scene pair for a subsequent memory test. Each study block was immediately followed by the corresponding test block, which consisted of 12 trials. Each test trial began by presenting a previously studied scene on the screen for 3000 msec (*scene cue*). After seeing the scene, participants made a FOK judgment on an 11-point percentage scale ranging from 0 to 100 in intervals of 10. Participants were instructed that a rating of 0 meant they were “absolutely certain” that they would not recognize the correct face, a rating of 100 meant they were “absolutely certain” that they would recognize the correct face, and ratings from 10 to 90 indicated intermediate levels of certainty. Participants were encouraged to use the entire scale. This was followed by a gaze-contingent fixation for 500 ms, to ensure that participants began the 3AFC recognition test with eye position in the same place and equidistant from each of the three alternatives. Once a fixation was measured, three faces were superimposed on the scene, and subjects were asked to indicate, via button press, which face had been paired with the scene during the study phase. One of the faces was correct and had been previously paired with the scene, whereas the other two had been previously paired with other scenes. Thus each face was familiar to the participants, and the recognition task could not be solved on familiarity alone. The correct face appeared in the left, right, and bottom position an equal number of times across each block. Participants had a maximum on 10000 ms to indicate their recognition response. After 10000 ms or the button press indicating their choice, the trial advanced and participants made a RCJ, once again, using an 11-point percentage scale ranging from 0 to 100 in intervals of 10, to indicate how certain they were that they chose the correct face. Note that each studied face was only viewed once during the test block, resulting in one–third of the studied face-scene pairs being tested. The face-scene pairs were counterbalanced across participants such that each face-scene pair was tested equally often. Although this paradigm deviated from the typical recall-judgment-recognition paradigm used to test FOKs as it did not test for recall of the specific face paired with the scene, because faces are hard to verbally label, we chose to use it because it has been well characterized in terms of eye movement based memory effects related to confidence and accuracy (Hannula et al., 2007; Hannula and Ranganath, 2009; Richmond and Nelson, 2009; Chua et al., 2012).

### Eye Tracking Acquisition and Analysis

Eye position was measured using an SMI iView RED eye tracker (SensoMotoric Instruments, Teltow, Germany) controlled by iView X version 2.5 software (SensoMotoric Instruments) that recorded binocularly at a rate of 60 Hz. Prior to the presentation of the study items, participants’ eye position was calibrated using five-point calibration plus validation. If the error in the estimated position was greater than 0.75° of visual angle, the experiment was stopped and calibration restarted.

We previously used the number of fixations during the scene cue at test as an indirect measure of cue fluency, with fewer fixations indexing greater fluency (Chua et al., 2012). Unlike Chua et al. (2012), in the present study we recorded eye movements during the study phase, enabling us to calculate the reprocessing effect. Therefore, we also conducted analyses using a measure of cue fluency based on the reprocessing effect (Althoff and Cohen, 1999; Ryan et al., 2000), and indexed cue fluency based on the difference in the number of fixations during the scene cue at study and the scene cue at test. This measure may better capture the change in fluency for a particular scene from study to test, with a bigger drop in fixations from study to test indicating a bigger change in fluency. Furthermore, it helps account for variation in the number of fixations due to stimulus differences. We analyzed data using both the total number of fixations and the difference in fixations from study to test. Because the analyses showed similar results, we only report results using the *difference in fixations from study to test* as our measure of cue fluency. Fixations were calculated oﬄine using SMI’s BeGaze 3.5 Software (Teltow, Germany).

For the 3AFC recognition test, we took an area of interest approach (AOI), and examined viewing behavior among four different AOIS: the three faces and the scene. For each face, we characterized the AOI as the *Correct Face, Incorrect Face 1*, and *Incorrect Face 2*. We also characterized an additional face AOI as the *Chosen Face*, which was the face indicated via button press by the participant during the recognition task. For hits the chosen face was the correct face, and for misses the chosen face was the incorrectly selected face (i.e., if the correct answer was in position ‘1’ and the participant indicated ‘2,’ the chosen face would be ‘2’). Our primary face AOIs of interest were the Correct Face and the Chosen Face, and viewing directed toward these faces were analyzed separately. Analyses of viewing directed to the correct face provides information about trace access regardless of behavioral response (Hannula et al., 2007; Richmond and Nelson, 2009), whereas viewing directed to the chosen face can reveal differences in viewing behavior related to the recognition decision, and also reveal memory effects above and beyond choice (Hannula et al., 2007; Hannula and Ranganath, 2009). For our purposes, the proportion of viewing directed at the chosen face can reveal information about the recognition decision. For example, we would expect that participants would spend more time viewing faces selected with high confidence, whereas for lower confidence responses we would expect participants to direct viewing more evenly among the choices, resulting in a lower proportion of viewing to the chosen face. We calculated the *proportion of viewing* directed to each AOI based on fixation durations as an overall metric of viewing behavior.

Previous research using a similar face-scene associative memory paradigm has indicated that rapid eye movements to the target face are obligatory and provide evidence of the previously encoded association (Hannula et al., 2007). Therefore, in addition to proportion of viewing directed at each AOI, we also examined how rapidly participants fixated within the AOI and analyzed whether the *onset of the first fixation* in the AOI differed by FOK, RCJ, or recognition accuracy.

### Data Analysis

Two-tailed paired *t*-tests were used to examine task performance in terms of recognition and metamemory performance. Although participants were instructed to make metamemory judgments on a scale ranging from 0 to 100, the actual scale was an 11-point percentage scale ranging from 0 to 100 in intervals of 10, and thus metamemory judgments were analyzed on the 11-point scale.

We analyzed the relative accuracy of FOKs and RCJs in two ways: using the Goodman–Kruskal gamma correlation and using *d*_a_, a measure derived from signal-detection theory. We calculated the Goodman–Kruskal gamma coefficient because, historically, it has been the most commonly used correlation to measure metamnemonic accuracy (Nelson, 1984; Gonzalez and Nelson, 1996). Gamma values range from -1 to 1, with 1 indicating that metamemory judgments perfectly predict accuracy, 0 indicating no correlation, and -1 indicating that metamemory judgments negatively predict accuracy. However, gamma has been criticized and may be less optimal than other measures because: (1) it treats metamnemonic judgments as an ordinal measure, thus making it insensitive to differences in the magnitude of the judgments (Masson and Rotello, 2009), (2) it has very low levels of stability across split halves and alternative forms of tests (Nelson, 1988; Thompson and Mason, 1996), and (3) it does not allow for interval-level interpretations of the data, which is necessary to accurately assess between group manipulations or interactions (Benjamin and Diaz, 2008). Recent research has suggested the use of *d*_a_, a measure derived from the signal-detection framework may be superior to gamma (Benjamin and Diaz, 2008; Toth et al., 2011). Therefore, we also computed *d*_a_, a measure based in signal detection theory. To compute *d*_a_, we used the procedure described by Benjamin and Diaz (2008) and used the formula *d*_a_ = √2*y_0_*/(1 + *m*^2^) where *y*_0_ and *m*^2^ represent the y intercept and slope, respectively, of a normal deviate isosensitivity function. Some researchers use a similar SDT derived statistic, *d′*, on metamemory judgments (sometimes referred to as Type 2 decisions, resulting in Type 2 *d′*) as a measure of metamnemonic accuracy (Fleming and Lau, 2014). Both *d′* and *d*_a_ can be conceptualized as distance based measures, and thus range from +∞ to -∞, with zero representing chance performance. Unlike *d*_a_, *d′* assumes common variance of the underlying distributions—an assumption that is often found to be incorrect (Swets, 1986). Still, *d′* is commonly used because it can be calculated when the rating scale has as few as two discrete choices. At least three discrete choices must be used for calculating *d*_a_.

#### Relating Eye Movement Data and Metamemory

As a first step, we did two sets of multi-level models, examining whether different eye movement measures covaried with (1) FOKs and (2) RCJs. As a subsequent analysis step, we examined whether specific covariates were still significant predictors of one metamemory judgment, when controlling for the other metamemory judgment (i.e., RCJs were included as a covariate in the FOK models and FOKs were included as a covariate in the RCJ models).

We used multi-level modeling in SPSS 22.0 to model both trial level and subject level variability in FOKs and RCJs. Trials in which subjects failed to provide a button response were excluded. Subjects and stimuli were treated as random effects with a varying intercept (Judd et al., 2012). All other effects were fixed effects. Recognition Accuracy was entered as a factor in the model (hits = 1 and misses = 0). Continuous variables (e.g., eye movement measures, metamemory ratings) were mean centered at the subject level, and entered as covariates. Models were estimated using Maximum Likelihood Estimation. Models were compared using likelihood ratio tests. Significant two-way interactions were followed up using simple slope tests (Aiken et al., 1991; Dawson, 2013). The simple slopes were evaluated for hits and misses (values 0 and 1, respectively) and 1 SD above or below the mean of the independent variable.

In one case, we used mediation analysis on our multilevel data, taking an approach that combines the dependent variable and mediator into a stacked variable and then uses that in the multilevel model (Bauer et al., 2006). We used the mixed procedure in SPSS, to obtain values for the indirect effect of the mediator on the dependent variable. To assess the mediation, a Monte Carlo resampling method was used with 20000 simulations to obtain 95% Confidence Intervals for the indirect effects using an R web utility (Preacher and Selig, 2010).

## Results

### Task Performance

#### Memory Performance

Participants (*N* = 66) performed well on the recognition task, choosing the correct face 71% ± 0.02% of the time (Mean ± SEM).

#### Metamemory Performance

First we computed the mean FOK and RCJ ratings for both hits and misses. These data show that both FOKs and RCJs were meaningfully related to memory in that participants gave higher ratings (scale 0–10) for hits than misses, for both FOKs [FOK for hits: 6.77 ± 0.18, FOK for misses: 5.51 ± 0.20, *t*(65) = 10.70, *p* < 0.00001, 95% CI of the difference for hits and misses (1.02,1.49)] and RCJs [RCJ for hits: 7.60 ± 0.16, RCJ for misses: 5.05 ± 0.19, *t*(65) = 15.21, *p* < 0.00001, 95% CI of the difference for hits and misses (2.21,2.88)]. However, consistent with the idea that metamnemonic judgments are made online and depend on different inputs at different times over the course of retrieval, compared to FOKs, RCJs were higher for hits [*t*(65) = 9.67, *p* < 0.00001, 95% CI of the difference between FOKs and RCJ for hits (0.66,1.0)] and lower for misses [*t*(65) = 3.38, *p* < 0.001, 95% CI of the difference between FOKs and RCJ for misses (0.19,0.73)]. Furthermore, the difference between the mean metamnemonic judgments for hits and misses was greater for RCJs than for FOKs [mean difference for RCJs: 2.59 ± 0.17, mean difference for FOKs: 1.29 ± 0.12, *t*(65) = 9.67, *p* < 0.00001, 95% CI of the difference between FOKs and RCJs (1.04,1.58)], showing that RCJs made after retrieval better reflected the true difference between hits and misses than did FOKs elicited prior to retrieval.

The mean ratings provide compelling evidence that RCJs better reflected true memory than did FOKs, however, that analysis considers groups of items and does not capture the accuracy of metamnemonic judgments at the item-by-item level. Traditionally, the gamma correlation has been used to measure relative metamnemonic accuracy, that is, the extent to which individuals’ metamnemonic judgments reflect their own memory performance for one item relative to another. FOKs and RCJs were reasonably accurate, as shown by gammas (FOK: 0.43 ± 0.03; RCJ: 0.61 ± 0.03), and RCJs were more accurate than FOKs [*t*(65) = 7.05, *p* < 0.00001, 95% CI of the difference between FOKs and RCJs (0.13,0.23)]. Similar to gamma, FOKs and RCJs were reasonably accurate, as shown by *d*_a_ (FOK: 0.62 ± 0.057; RCJ: 1.12 ± 0.072)^1^, and RCJs were more accurate than FOKs, as measured by *d*_a_ [*t*(64) = 9.14, *p* < 0.00001, 95% CI of the difference between FOKs and RCJs (0.40,0.62)].

#### Metamemory Judgments Over Time

One way to examine metamemory across the learning and memory time scale is to examine the difference in FOKs and RCJs. As one would expect, metamemory judgments change with additional mnemonic experience (i.e., retrieval attempts, weighing of alternatives, and making a recognition decision). Examination of trial-by-trial changes in ratings (FOK–RCJ) showed that the recognition experience led to a change in ratings [*t*(65) = -9.69, *p* < 0.00001, 95% CI of FOK–RCJ (-1.55,-1.02)]; for hits, the level of RCJs were 0.83 ± 0.09 higher than FOKs, whereas for misses, RCJs were 0.46 ± 0.14 lower than FOKs.

The change in ratings makes it clear that our subjective evaluation of our memory changes with more mnemonic experience, but it is also possible that prior metamemory judgments could influence subsequent metamemory judgments. To test this, we used multilevel modeling to examine FOK rating as a predictor of RCJs, and compared the addition of this covariate to a model without it. In the first model, we entered recognition accuracy and reaction time, and their interaction, as covariates. As expected, recognition accuracy and speed of retrieval were significant predictors of RCJs (**Table 1**). To test whether FOK ratings also influenced RCJs, we added FOK rating to the model. FOK rating was also a significant predictor of RCJs (**Table 1**). Furthermore, a likelihood ratio test showed that the model including FOK ratings fit better than the one without FOK ratings [χ^2^(1) = 777, *p* < 0.00001]. Thus, individuals’ prospective metamemory judgments, combined with their retrieval experience, predict RCJs.

**Table 1:** Multi-level modeling of retrospective confidence judgments (RCJ).

Model	Parameter	Estimate	SE	∼df	*t*	-2LL
1	Accuracy	-2.00	0.09	3432	-21.39^∗∗∗^	15504
	RT	-0.79	0.35	3373	-22.75^∗∗∗^	
	Accuracy × RT	0.37	0.066	3399	5.58^∗∗∗^	
2	Accuracy	-1.51	0.085	3424	-17.79^∗∗∗^	14727
	RT	-0.52	0.032	3359	-16.08^∗∗∗^	
	Accuracy × RT	0.12	0.06	3405	2.08^∗^	
	FOK rating	0.48	0.016	3323	29.74^∗∗∗^	

*Accuracy, Recognition accuracy; RT, Reaction time; -2LL, -2 Restricted log likelihood.*

*^∗^*p* < 0.05, ^∗∗∗^*p* < 0.001.*

### Eye Movements

#### Fixations during the Scene Cue and Cue Fluency

Based on prior research suggesting that cue fluency contributes to FOKs (Schwartz and Metcalfe, 1992; Metcalfe et al., 1993; Koriat and Levy-Sadot, 2001) and RCJs (Koriat et al., 2008; Chua et al., 2012), we capitalized on the *reprocessing effect* (Althoff and Cohen, 1999; Ryan et al., 2000) and used the difference in the number of fixations to the scene cue at study and test to examine cue fluency. We used multi-level modeling to examine whether cue fluency covaried with (1) FOKs and (2) RCJs (**Table 2**). The model also included recognition accuracy and the accuracy × cue fluency interaction. Cue fluency was related to higher FOKs (*p* < 0.001) and RCJs (*p* < 0.001), and there was no significant interaction with accuracy (FOKs: *p* > 0.9; RCJs: *p* > 0.2). For FOKs, cue fluency was still a significant predictor (*p* < 0.001), even when controlling for RCJs, suggesting that cue fluency makes a direct contribution to FOKs. However, for RCJs, cue fluency was no longer a significant predictor (*p* > 0.2) when controlling for FOKs, suggesting that the influence of cue fluency on RCJs may be indirect and occur via prospective metamemory judgments (**Table 2**).

**Table 2:** Multi-level modeling of metamemory judgments by cue fluency (measured as the number of fixations to the scene cue during study minus the number of fixations to the scene cue at test) and recognition accuracy.

		Feeling-of-knowing (FOK) rating					Retrospective confidence judgment (RCJ)				
Model	Parameter	Estimate	SE	∼df	*t*	-2LL	Estimate	SE	∼df	*t*	-2LL
1	Accuracy	-1.08	0.086	3404	-12.5^∗∗∗^	15412	-2.32	0.095	3426	-24.6^∗∗∗^	16109
	Cue fluency	0.078	0.017	3340	4.62^∗∗∗^		0.063	0.018	3354	3.42^∗∗∗^	
	Accuracy × Cue fluency	0.004	0.031	3343	0.117		-0.040	0.034	3361	-1.18	
2	Accuracy	-0.050	0.081	3403	-0.622	14438	-1.74	0.084	3422	-20.93^∗∗∗^	15032
	Cue fluency	0.053	0.015	3368	3.59^∗∗∗^		0.019	0.016	3381	1.18	
	Accuracy × Cue fluency	0.018	0.027	3371	0.688		-0.036	0.029	3390	-1.23	
	FOK						0.55	0.016	3309	34.15^∗∗∗^	
	RCJ	0.46	0.014	3385	33.84^∗∗∗^						

*Accuracy, Recognition accuracy; -2LL, -2 Restricted log likelihood.*

*^∗∗∗^*p* < 0.001.*

**FIGURE 2:**
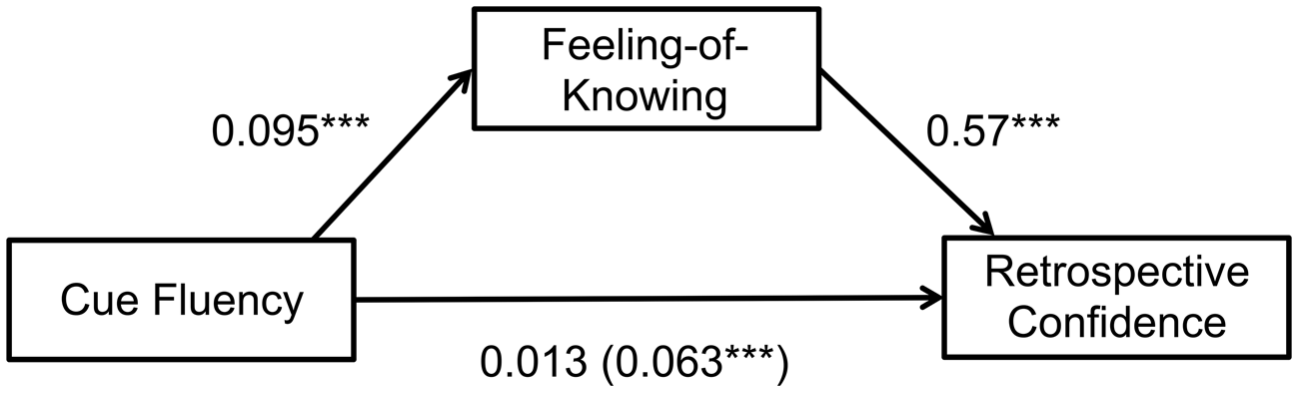
**Feeling-of-Knowing (FOK) judgments mediate the effect of cue fluency, as measured by the change in fixations to the scene cue from study to test, on retrospective confidence judgments (RCJs).** Value in parentheses represents the effect of scene fixations on retrospective confidence when FOKs are not modeled as a mediator. ^∗∗∗^*p* < 0.0001.

To test the idea that the effects of cue fluency on RCJs are mediated by FOKs, we ran a mediation analysis. When FOK was included as a mediator, the effect of cue fluency on RCJs became non-significant [*b* = 0.013 ± 0.015, t(2987) = 0.90, *p* > 0.35; **Figure 2**]. The indirect effect was significant, with FOKs mediating the effect of cue fluency on RCJs (ab: 0.054 ± 0. 0086, 95%CI of ab 0.038, 0.072, Z = 6.34, *p* < 0.0001).

#### Viewing Directed to the Correct Face and Target Accessibility

Target accessibility has been thought to subserve both FOKs (Koriat, 1993, 1994, 1995) and RCJs (Koriat, 2008b, 2012), and we examined this using two eye movement measures: overall proportion of viewing time directed to the correct face and the onset of the first fixation to the correct face. We used multi-level modeling to examine whether each variable covaried with (1) FOKs and (2) RCJs (**Table 3**). Each model also included recognition accuracy and the accuracy × eye movement interaction.

**Table 3:** Multi-level modeling of metamemory judgments by viewing directed to the correct face and recognition accuracy.

		Feeling-of-knowing (FOK) rating					Retrospective confidence judgment (RCJ)				
Model	Parameter	Estimate	SE	∼df	t	-2LL	Estimate	SE	∼df	*t*	-2LL
1	Accuracy	-0.89	0.10	3384	-8.55^∗∗∗^	15405	-2.15	0.11	3415	-18.91^∗∗∗^	15955
	PropVT correct	1.66	0.28	3358	5.87^∗∗∗^		2.70	0.31	3377	8.75^∗∗∗^	
	Accuracy × PropVT correct	-0.63	0.56	3342	-1.13		-2.33	0.61	3366	-3.83^∗∗∗^	
2	Accuracy	0.051	0.096	3389	0.54	14454	-1.68	0.10	3424	-16.81^∗∗∗^	14988
	PropVT correct	0.44	0.25	3378	1.78		1.82	0.27	3395	6.78^∗∗∗^	
	Accuracy × PropVT correct	0.41	0.49	3367	0.85		-2.08	0.53	3395	-3.93^∗∗∗^	
	FOK						0.54	0.016	3284	33.79^∗∗∗^	
	RCJ	0.46	0.014	3387	33.37^∗∗∗^						
3	Accuracy	-1.10	0.086	3399	-12.74^∗∗∗^	15438	-2.33	0.095	3424	-24.65^∗∗∗^	16030
	First fix ons correct	-0.00021	0.000090	3365	-2.34^∗^		-0.00012	0.00010	3379	-1.26	
	Accuracy × First fix ons Correct	0.00026	0.00015	3321	1.79		0.00016	0.00016	3338	0.99	
4	Accuracy	-0.066	0.081	3398	-0.81	14456	-1.73	0.084	3421	-20.70^∗∗∗^	15034
	First fix ons correct	-0.00016	0.000078	3388	-2.10^∗^		0.000001	0.000085	3390	0.013	
	Accuracy × First fix ons correct	0.00019	0.00013	3351	1.48		-0.000003	0.00014	3374	0.021	
	FOK						0.55	0.016	3292	34.37^∗∗∗^	
	RCJ	0.46	0.014	3385	34.00^∗∗∗^						

*Accuracy, Recognition accuracy; -2LL, -2 Restricted log likelihood; PropVT, proportion of viewing; First fix ons, onset of the first fixation.*

*^∗^*p* < 0.05, ^∗∗∗^*p* < 0.001.*

**FIGURE 3:**
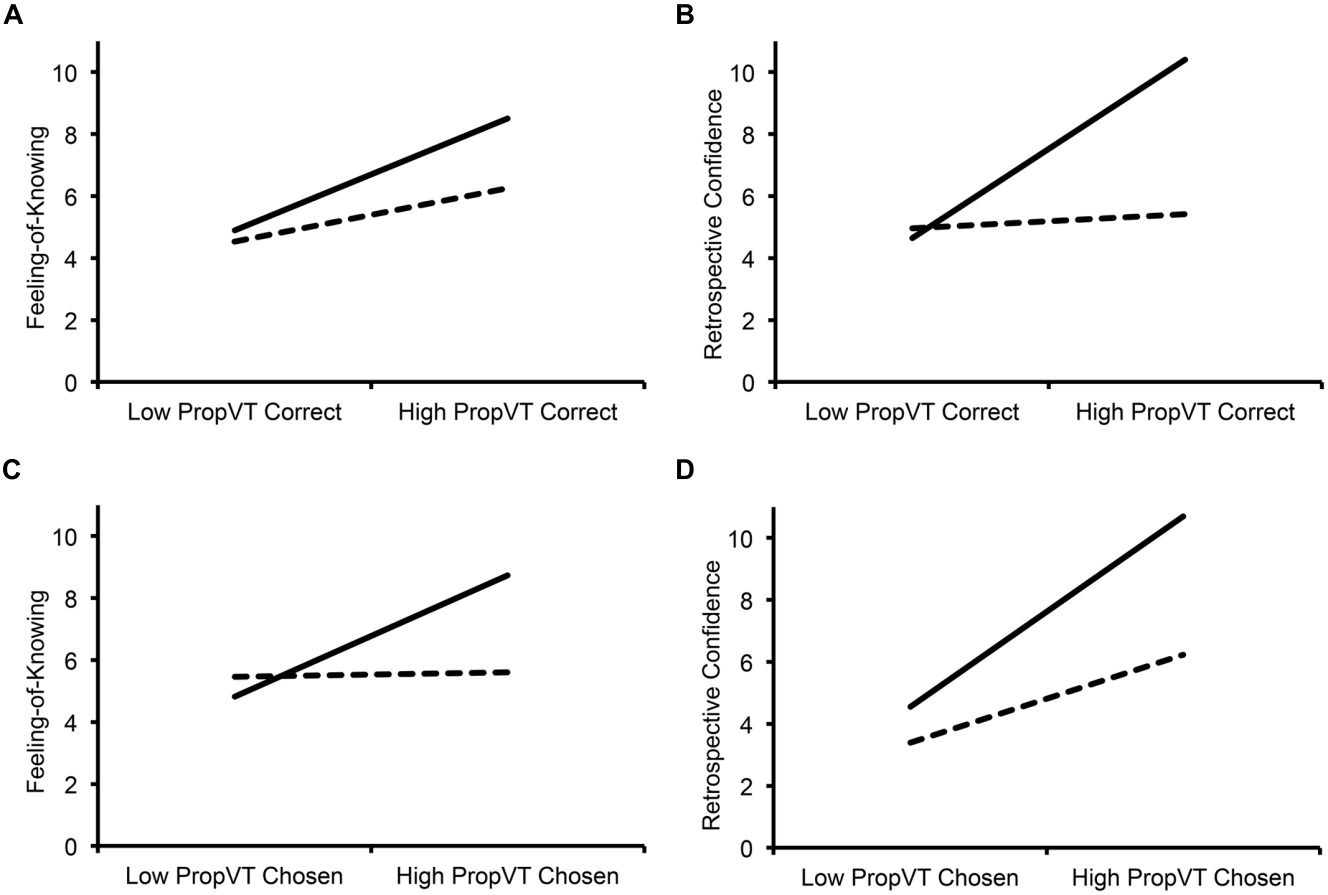
**The estimated relationship between proportion of viewing to the correct/chosen face and metamemory judgments.** Correct responses are shown in solid lines and incorrect responses in dashed lines. A higher proportion of viewing directed to the correct face was associated with higher FOK judgments for correct and incorrect recognition **(A)**, but was associated with higher RCJs for correct recognition only **(B)**. In contrast, a higher proportion of viewing directed to the chosen face was associated with higher FOK judgments for correct recognition only **(C)**, but was associated with higher RCJs for correct and incorrect recognition **(D)**. PropVT Correct = Proportion of Viewing Time directed to the Correct Face; PropVT Chosen = Proportion of Viewing Time directed to the Chosen Face.

We first examined the relationship between proportion of viewing directed to the correct face and FOKs (**Table 3**; **Figure 3**). Consistent with the hypothesis that FOKs are related to target accessibility regardless of whether subsequent recognition is accurate or not, a higher proportion of viewing directed to the correct face was associated with higher FOKs for correct and incorrect recognition (*p* < 0.001), and there was no significant interaction with accuracy (*p* > 0.25; **Figure 3A**). To determine whether this effect remained when controlling for RCJs, we ran a subsequent model including RCJs as a covariate; there was only a trend for higher proportion of viewing directed to the correct face associating with higher FOKs (*p* < 0.08).

We also examined the relationship between onset of the first fixation to the correct face and FOKs (**Table 3**), reasoning that the speed of memory-based attentional capture indexed target accessibility (Hannula et al., 2007; Richmond and Nelson, 2009). Like the proportion of viewing to the correct face, analyses of onset of the first fixation to the correct face were consistent with the hypothesis that FOKs are related to target accessibility. Faster onsets of the first fixation to the correct face were associated with higher FOKs for correct and incorrect recognition (*p* < 0.05), and there was no significant interaction with accuracy (*p* > 0.07). As shown by subsequent models that included RCJs as a covariate, faster onsets of the first fixation to the correct face was still associated with higher FOKs when controlling for RCJs (*p* < 0.05; **Table 3**), and there was no interaction with accuracy (*p* > 0.1).

We next examined the relationship between proportion of viewing directed to the correct face and RCJs (**Table 3**; **Figure 3B**). Demonstrating that RCJs for correct and incorrect recognition have different relationships to target accessibility (**Figure 3B**), there was a significant accuracy × proportion of viewing directed to the correct face interaction (*p* < 0.001) such that increased viewing led to higher RCJs for correct recognition [*B* = 2.70; *t*(3451) = 4.87, *p* < 0.001], but not incorrect recognition [*B* = 0.370; *t*(3451) = 0.435, *p* > 0.65]. As shown by subsequent models that included FOKs as a covariate, this effect remained when controlling for FOKs (*p* < 0.001; **Table 3**). Also consistent with the idea that RCJs are not based on target accessibility, the onset of the first fixation to the correct face was not a significant predictor of RCJs (*p* > 0.2), nor was its interaction with accuracy (*p* > 0.3; **Table 3**).

#### Viewing Directed to the Chosen Face

To determine the influence of the ease of the recognition decision on FOKs and RCJs, we examined the proportion of viewing directed to the chosen face and the onset of the first fixation to the chosen face (**Table 4**). The chosen face was the face that the subject indicated via button press to be the face that was originally paired with the scene. It is worth noting that for correct recognition, the chosen face, and the correct face are the same, whereas for incorrect responses, the chosen face was an incorrect face. Thus the values for the proportion of viewing and onset of the first fixation directed to the correct and chosen face are the same for correct recognition, but not incorrect recognition. Therefore, the added value of examining how these viewing measures directed to the chosen face relate to metamemory judgments is for incorrect recognition.

**Table 4:** Multi-level modeling of metamemory judgments by viewing directed to the chosen face and recognition accuracy.

		Feeling-of-knowing (FOK) rating					Retrospective confidence judgment (RCJ)				
Model	Parameter	Estimate	SE	∼df	*t*	-2LL	Estimate	SE	∼df	*t*	-2LL
1	Accuracy	-1.08	0.87	3402	-12.40^∗∗∗^	15404	-2.24	0.095	3427	-23.61^∗∗∗^	15932
	PropVT chosen	1.78	0.28	3354	6.27^∗∗∗^		2.88	0.31	3371	9.36^∗∗∗^	
	Accuracy × PropVT chosen	-1.76	0.53	3346	-3.30^∗∗∗^		-1.07	0.58	3366	-1.85	
2	Accuracy	-0.10	0.082	3402	-1.18	14452	-1.66	0.084	3421	-19.71^∗∗∗^	14964
	PropVT chosen	0.49	0.25	3374	1.96^∗^		1.95	0.27	3391	7.23^∗∗∗^	
	Accuracy × PropVT chosen	-1.30	0.46	3372	-2.80^∗∗^		-0.15	0.50	3394	-0.29	
	FOK						0.54	0.16	3282	33.79^∗∗∗^	
	RCJ	0.46	0.014	3389	33.38^∗∗∗^						
3	Accuracy	-1.10	0.086	3402	-12.74^∗∗∗^	15437	-2.33	0.094	3426	-24.68^∗∗∗^	16029
	First fix ons chosen	-0.00022	0.000090	3363	-2.46^∗^		-0.00013	0.00010	3376	-1.35	
	Accuracy × First fix ons chosen	0.000085	0.00016	3350	0.55		0.000016	0.00017	3366	0.092	
4	Accuracy	-0.066	0.081	3400	-0.81	14456	-1.73	0.084	3421	-20.74^∗∗∗^	15034
	First fix ons chosen	-0.00017	0.000078	3387	-2.18^∗^		-0.000002	0.000085	3389	-0.025	
	Accuracy × First fix ons chosen	0.000090	0.00014	3378	0.51		-0.000046	0.00015	3393	-0.31	
	FOK						0.55	0.016	3293	34.35^∗∗∗^	
	RCJ	0.46	0.014	3385	33.98^∗∗∗^						

*Accuracy, Recognition accuracy; -2LL, -2 Restricted log likelihood; PropVT Chosen, proportion of viewing to the chosen face; First fix ons Chosen, onset of the first fixation to the chosen face.*

*^∗^*p* < 0.05, ^∗∗^*p* < 0.01, ^∗∗∗^*p* < 0.001.*

For FOKs (**Table 4**; **Figure 3C**), there was a significant viewing directed to the chosen face × accuracy interaction (*p* < 0.001) such that a higher proportion viewing of the chosen face was associated with higher FOKs for correct recognition [*B* = 1.78; *t*(3451) = 3.34, *p* < 0.001], but not incorrect recognition [*B* = 0.021; *t*(3451) = 0.026, *p* > 0.95; **Figure 3C**]. This interaction remained significant when controlling for RCJs (*p* < 0.005). This suggests that recognition choice does not significantly relate to FOKs overall, and is consistent with the idea that FOKs are related to target accessibility (see section Viewing Directed to the Correct Face and Target Accessibility) rather than accessibility of any choice.

To further test whether FOKs were related to accessibility of any choice, we examined the relationship between onset of the first fixation to the chosen face and FOKs. Faster onsets of the first fixation to the chosen face were associated with higher FOKs (*p* < 0.05; **Table 3**), and did not interact with accuracy, and this remained significant when controlling for RCJs (*p* < 0.05). Unlike our previous analyses of the proportion of viewing directed to the chosen face, the finding that there is faster viewing of the chosen face, regardless of accuracy, is more consistent with partial access theories of FOK.

We also examined viewing directed to the chosen face for RCJs (**Table 4**; **Figure 3D**). The proportion of viewing directed to the chosen face was associated with higher RCJs overall (*p* < 0.001; **Figure 3D**), and did not significantly interact with accuracy (*p* > 0.06). When controlling for FOKs, the main effect, with increased viewing directed to the chosen face predicting higher RCJs for correct and incorrect recognition, remained significant (*p* < 0.001). Although overall proportion of viewing to the chosen face was a significant predictor of RCJs, the onset of the first fixation to the chosen face was not (*p* > 0.2). Nevertheless, the findings that increased viewing of the chosen face is associated with higher RCJs is consistent with the idea that recognition confidence is based, at least in part, on the ease of decision-making and choice behavior.

#### Changes in Metamemory Judgments

Given that FOKs influence RCJs, one question that arises is what leads to changing one’s rating of certainty after making that first metamemory judgment. That is, if you’ve given an FOK of 7, what would happen during recognition that would lead to lowering one’s confidence to a 5, maintaining one’s confidence at a 7, or raising it to a 10. To examine this we constructed a model with the change in metamemory rating (FOK–RCJ) as the dependent variable, and included different factors related to target accessibility and the recognition decision as predictors (**Table 5**). In one model, we focused on viewing directed to the correct face to examine target accessibility, and entered the proportion of viewing directed to the correct face, its interaction with accuracy, the onset of the first fixation to the correct face, its interaction with accuracy, the initial FOK rating, and recognition accuracy in the model. In addition to accuracy (*p* < 0.001) and FOKs (*p* < 0.001) being significant predictors of the change in metamemory rating, increased viewing of the correct face interacted with accuracy such that for hits it led to raising one’s RCJ higher than the FOK [*B* = -2.32, *t*(3448) = -4.34, *p* < 0.001] and for misses it had no effect [*B* = 0.28, *t*(3448) = 0.33, *p* > 0.7]. There was also an interaction of first fixation onset to the correct face and accuracy (*p* < 0.05), but the simple slopes were not significant. Thus, more accurate confidence judgments (i.e., those where higher confidence ratings are given to hits) are based on target accessibility, whereas less accurate confidence ratings (i.e., those where higher confidence ratings are given to misses) are not.

**Table 5:** Multi-level modeling of change in metamemory judgments by viewing directed to correct/chosen face and recognition accuracy.

		Change in rating (FOK–RCJ)				
Model	Parameter	Estimate	SE	∼df	*t*	-2LL
1	Accuracy	1.63	0.10	3448	16.36^∗∗∗^	14893
	FOK	0.45	0.016	3277	28.27^∗∗∗^	
	PropVT correct	-2.32	0.30	3409	-7.77^∗∗∗^	
	First fix ons corr	-0.00032	0.000094	3402	-3.43^∗∗∗^	
	Accuracy × PropVT correct	2.60	0.57	3428	4.58^∗∗∗^	
	Accuracy × First fix ons correct	0.00035	0.00015	3400	2.31^∗^	
2	Accuracy	1.62	0.083	3454	19.39^∗∗∗^	14867
	FOK	0.46	0.016	3279	28.47^∗∗∗^	
	PropVT chosen	-2.39	-0.30	3393	-7.99^∗∗∗^	
	First fix onset chosen	-0.00033	0.000094	3397	-3.48^∗∗∗^	
	Accuracy × PropVT chosen	0.31	0.54	3405	0.56	
	Accuracy × First fix ons chosen	0.00016	0.00016	3403	1.00	

*Accuracy, Recognition accuracy; FOK, Feeling-of-knowing; RCJ, Retrospective confidence judgment; -2LL, -2 Restricted log likelihood; PropVT correct, proportion of viewing to the correct face; First fix ons correct, onset of the first fixation to the correct face; PropVT chosen, proportion of viewing to the chosen face; First fix ons Chosen, onset of the first fixation to the chosen face.*

*^∗^*p* < 0.05, ^∗∗∗^*p* < 0.001.*

We also examined the possibility that information related to the recognition decision was driving the change in confidence for both hits and misses, and therefore, we focused on viewing directed to the chosen face. We constructed a second model with the change in metamemory rating (FOK–RCJ) as the dependent variable, and entered the proportion of viewing directed to the chosen face, its interaction with accuracy, the onset of the first fixation to the chosen face, its interaction with accuracy, the initial FOK rating, and recognition accuracy in the model. In addition to accuracy (*p* < 0.001) and FOKs (*p* < 0.001) being significant predictors, increased (*p* < 0.001) and faster (*p* < 0.001) looking at the chosen face predicted raising ones confidence above one’s FOK, with no interaction with accuracy (**Table 5**). The consistent association of viewing directed to the chosen face with raising one’s confidence above the initial FOK is consistent with the idea that, in addition to target accessibility, the recognition decision-making experience is driving confidence and, unlike target accessibility, does so regardless of accuracy.

## Discussion

To examine how memory monitoring changes over time, and the basis for those changes, we used eye movement indices of memory to examine how cue fluency, target accessibility, and choice behavior influence (1) FOKs, and (2) RCJs. We showed that early metamemory judgments, namely FOKs, are based on cue fluency and accessibility. Later metamemory judgments, namely RCJs, are based on the decision-making experience, and the earlier metamemory judgment.

### Feeling-of-Knowing Judgments: Cue Fluency and Target Access

After viewing a cue, but before the 3AFC recognition test, participants were asked to indicate their certainty about their future recognition performance by indicating their FOK. Consistent with prior research showing that cue-related processing influences FOKs (Schwartz and Metcalfe, 1992; Metcalfe et al., 1993), we showed that a greater change in fixations to the scene cue from study to test, which indexes more fluent processing, was related to higher FOKs.

A more controversial basis of FOKs relates to accessibility. Although early models of FOKs proposed that they were based on direct access to the target (Metcalfe, 2000), there is evidence against such an account of FOKs (Koriat, 2000; Koriat and Levy-Sadot, 2000). Here, we used eye movements directed to the correct face as an indirect measure of direct access to the target. Rapid viewing of the correct face is thought to be an obligatory effect of memory on eye movements (Hannula et al., 2007; Ryan et al., 2007), and higher FOKs were associated with faster fixations to the correct face, for both correct and incorrect responses. Although our measure of target access is indirect, this is consistent with the idea that for higher FOKs subjects had access to the target, and this led to faster fixations to the target. Thus, it appears that direct access can serve as a basis of FOKs.

However, we also showed that faster fixations to the incorrectly chosen face for misses predicted higher FOKs, which is difficult for direct access theories to explain. This kind of illusory FOK, could be based on partial access to the target, but could also be based on erroneous, yet accessible information. For example, after viewing a scene cue, a participant might recall that the target has red hair and, as a result, give a high FOK, and look more quickly to a target with red hair, even if the correct face has brown hair. In such a case, the FOK appears to be based on the amount or fluency of accessible information, which is different than direct access to the target (Koriat, 1995). Thus it appears that to make FOKs, individuals may monitor accessibility without respect to accuracy (Koriat, 1995, 2000; Koriat and Levy-Sadot, 2001). However, not all accessible features will increase FOKs; the information must be relevant and meaningful (Thomas et al., 2012). For example, if a person recognizes a scene, they may recall they imagined the individual acting in the scene, “I remember imagining the face picking the corn in the field,” but an individual may not use this information as the basis of their FOK if they recognize that remembering the action does not indicate whether they will remember the face. Overall, it appears that FOKs are based on accessibility of information, but this access does not necessarily have to be accurate.

One consideration for our findings is that typical FOK paradigms use a Recall-Judgment-Recognition format (Hart, 1965), and FOKs are made about unrecallable items only (Nelson and Narens, 1990). In our paradigm, we did not explicitly test for recall, and participants made a FOK judgment after every trial. Because participants were presented with a scene cue, and likely attempted to recall the associated face, we may have some trials in which participants recalled the face associated with the scene. This could explain why we get faster fixations to the target face. Although this poses some limitation for comparisons with other studies, it is unlikely to change our interpretation of the basis for FOKs. First, our analyses were based on continuous variables ranging from 0 to 10, and tested for linear effects, so it is unlikely that recalled items, which presumably would be given the highest FOK rating, would alter our results. Second, because our findings are consistent for both correct and incorrect recognition, it seems unlikely that removing successfully recalled items would change the results.

### Retrospective Judgments: FOK and Choice Experience

Whereas most studies examine FOKs and RCJs in isolation, we examined metamemory judgments across time. Our results showed that FOKs predict RCJs, which suggests that a participant’s expectations about his or her performance influenced his or her confidence. At face value, FOKs appear to measure expected performance. Empirically, they have also been related to beliefs about what should be known (i.e., expectations) in the general knowledge domain (Costermans et al., 1992; Marquié and Huet, 2000). As one might expect given that FOKs predict confidence, general beliefs about ability have been shown to predict confidence in both general knowledge and eyewitness memory paradigms (Perfect, 2004). The effects of expectations on confidence have also been tested more directly. For example, using an episodic memory cueing paradigm, participants were told that a stimulus was either “Likely Old” or “Likely New,” and when this “Likely Old” cue was invalid, participants had decreased confidence in correct rejections (i.e., saying the item was new) compared to when the cue was “Likely New” (Jaeger et al., 2012). Thus, our findings that FOKs influence confidence are consistent with prior research showing that, in some cases, expectations can influence confidence.

The fact that expectations influence confidence may have relevance for findings showing that cue-related processing influences confidence (e.g., Koriat et al., 2008; Chua et al., 2012) because it suggests that the effect of cues on confidence may be indirect. Indeed, in our analyses, we initially showed that cue fluency predicted RCJs. However, subsequent mediation analyses revealed this was an indirect effect, with FOKs mediating the effect of cue fluency on confidence. This mediating effect is unlikely to rely on explicit FOK judgments; in a previous study, using a similar paradigm, but without FOK judgments, we showed that fixations to the scene cue predicted confidence (Chua et al., 2012). Similarly, general knowledge tasks, which show domain specific increases in confidence (Marquié and Huet, 2000), suggest that the effects of domain familiarity may be indirect in that participants give higher confidence ratings because they expect to know more in that domain.

The idea that expectations, or prospective metamemory judgments, can influence retrospective memory judgments may relate broadly to findings that metamemory judgments are inferential in nature (Schwartz et al., 1997; Koriat et al., 2008; Koriat and Ackerman, 2010). An FOK judgment may be another source of information that people use when making inferential RCJs. Work examining memory for deceptive and non-deceptive items, in which the deceptive information is based on gist or partial access has consistently shown that gist or partial access leads to higher retrospective confidence and decreased metacognitive accuracy (Brewer and Sampaio, 2006, 2012). What drives this effect is the inference that gist or partial access relates to accuracy. Because FOKs may also be based on partial access (Koriat, 1994), it is reasonable to think that participants may make the same inference that FOKs relate to accuracy.

Clearly, expectations are not the only basis for retrospective confidence; the recognition experience is a major contributor to confidence (Koriat et al., 2008; Koriat and Ackerman, 2010; Chua et al., 2012). This makes intuitive sense, and we also show that participants raise or lower their confidence after their recognition experience, and that RCJs are more accurate than FOKs (Watier and Collin, 2011). Furthermore, when we control for FOKs, the only covariates that significantly predict RCJs, or changing one’s metamemory rating, relate to the recognition experience. The variables that predicted confidence, and did not interact with accuracy, all related to choice behavior, namely the proportion of viewing time and the onset of the first fixation directed to the chosen face. Viewing directed to the correct face did not vary by confidence for incorrect recognition responses. Thus it appears that confidence comes from decision-making behavior rather than direct access to the memory trace alone. This is consistent with experience-based models of RCJs (Koriat et al., 2008) in that the online feedback about the recognition experience, as indexed by eye movements, predicted confidence, and changes in metamemory ratings. One recent experience-based model, the self-consistency model of confidence, proposes that confidence in forced choice tasks comes from the evidence for the choice based on the amount of conflict experienced when making a decision (Koriat, 2012), and our findings that confidence for correct and incorrect recognition tracks viewing of the chosen face is consistent with this.

Additional merits to the *self-consistency model of confidence* is that it may also explain the correlation between FOKs and RCJs. The model posits that confidence tracks reliability (Koriat, 2012). Reliability would come from consistent mnemonic information retrieved during the scene cue period, which would form the basis of the FOK, and the recognition task, which forms the basis of RCJs. To call on our earlier example, if the scene cue elicited a memory of a person with red hair, and there was a red haired individual on the three-face display, one might have higher confidence because there was consistency across time. If there were no individual with red hair in the three-face display, the participant might then drastically lower his or her confidence. Thus, an alternative explanation for why FOKs predict confidence is that confirmatory evidence over multiple time points leads to higher confidence (Koriat, 2012).

It is also possible that individual differences in motivation, beliefs, and biases could drive the relationship between FOKs and RCJs. One such bias may be the confirmation bias, which has some commonalities the self-consistency model of confidence (Koriat, 2012), in which individuals selectively look for information that confirm their hypotheses (Nickerson, 1998), and which there are known to be individual differences (Rassin, 2008). Similarly, individual differences in beliefs about one’s memory (Lineweaver and Hertzog, 1998; Magnussen et al., 2006) may also influence the degree to which participants are willing to update their confidence judgments based on the target retrieval experience. These beliefs may apply across the learning and memory timescale, and similarly bias individuals’ FOKs and RCJs. Our study did not involve any manipulation that might affect participants’ efforts to be consistent nor measure individual differences in biases, but this idea should be considered in future research.

Another reason that prospective and retrospective metamemory ratings may interact is that they share a common neural basis (Chua et al., 2009; Ryals et al., 2015). For example, recent fMRI work has shown that the act of making FOKs and RCJs shows common activation compared to other memory and non-memory tasks (Chua et al., 2009). Furthermore, experimental manipulation of brain activity by theta burst TMS over the frontopolar cortex selectively improved both prospective (in this case JOLs) and retrospective metamemory judgments (Ryals et al., 2015), indicating a shared neural basis for metamemory judgments that occur at different times across the learning and memory timescale.

It is worth mentioning that, in this study, viewing directed to the chosen face was indicative of confidence for both correct and incorrect recognition, unlike in our previous study (Chua et al., 2012), in which it tracked confidence for correct responses only. Differences in the paradigm and analyses may explain the discrepancy. In this study, we used a larger response scale and analyzed confidence as a continuous variable, whereas in the previous study we examined confidence as high, medium, and low. Additionally, in our previous study examined differences for a set period of time, whereas in this study, we examined viewing before the recognition response, which may have increased our sensitivity to detect effects.

## Conclusion

Memory monitoring is an ongoing process that that involves a dynamic model that changes across time. Memory monitoring assessed prior to recognition is based on cue fluency, and target accessibility leads participants to have expectations about their future performance. The experience during the recognition task, in particular the experience related to choice behavior, gives rise to subjective feelings of confidence in one’s answer. However, the target recognition experience only accounts for some of the variance, and one’s metamemory judgment prior to recognition also influences their metamemory judgment following recognition. These results indicate that metamemory judgments should not be thought of as distinct subjective experiences in time but as an evolving awareness that incorporates the past metamnemonic judgments with new information into a dynamic model of memory.

## Conflict of Interest Statement

The authors declare that the research was conducted in the absence of any commercial or financial relationships that could be construed as a potential conflict of interest.

## Footnotes

1 Data from 1 subject was not included in the analysis of *d_a_* because their data did not allow for the calculation of *d*_a_.
